# Pilot Study on the Impact of Biogas as a Fuel Source on Respiratory Health of Women on Rural Kenyan Smallholder Dairy Farms

**DOI:** 10.1155/2012/636298

**Published:** 2012-08-28

**Authors:** Carolyn Dohoo, Judith Read Guernsey, Kimberley Critchley, John VanLeeuwen

**Affiliations:** ^1^Department of Community Health & Epidemiology, Dalhousie University, 5790 University Avenue, Halifax, NS, Canada B3H 1V7; ^2^Centre for Veterinary Epidemiological Research, Department of Health Management, Atlantic Veterinary College, University of Prince Edward Island, 550 University Avenue, Charlottetown, PE, Canada C1A 4P3; ^3^School of Nursing, University of Prince Edward Island, 550 University Avenue, Charlottetown, PE, Canada C1A 4P3

## Abstract

Biomass burning in indoor environments has been highlighted as a major cause of respiratory morbidity for women and children in low-income countries. Inexpensive technological innovations which reduce such exposures are needed. This study evaluated the impact of low tech compost digesters, which generate biogas for cooking, versus traditional fuel sources on the respiratory health of nonsmoking Kenyan farmwomen. Women from 31 farms with biogas digesters were compared to age-matched women from 31 biomass-reliant farms, in June 2010. Only 43% of the biogas group reported any breathing problems, compared to 71% in the referent group (*P* = 0.03). Referent women self-reported higher rates of shortness of breath (52% versus 30%), difficulty breathing (42% versus 23%), and chest pain while breathing (35% versus 17%) during the last 6 months (*P* = 0.09 to 0.12) compared to biogas women. Biogas women demonstrated slightly better spirometry results but differences were not statistically significant, likely due to limited latency between biogas digester installation and spirometry testing. Most biogas women reported improved personal respiratory health (87%) and improved children's health (72%) since biogas digester installation. These findings suggest that using biogas in cookhouses improves respiratory symptoms but long-term impacts on lung function are unclear.

## 1. Introduction

**Figure 1:**
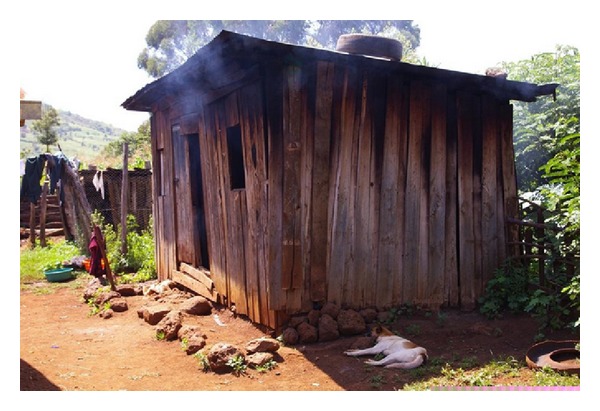
A typical rural Kenyan cookhouse with a small window for ventilation.

In rural Kenya, women regularly cook in poorly ventilated cookhouses, which are separate structures from their houses, using locally gathered wood as fuel (Figure 1). Consequently, women and young children in their care are often exposed to high levels of indoor air pollution, especially wood smoke [1]. The exposure to residential indoor air pollution in low-income countries has been identified as a major source of concern worldwide [2–4], with significant negative effects on the health of women and children, who typically spend more time indoors. The exposure to high levels of indoor air pollution associated with biomass burning (e.g., wood, crop residue, etc.) has been associated with respiratory health outcomes such as chronic obstructive pulmonary disease (COPD), respiratory infections, asthma, pneumonia, and tuberculosis [5–8]. A recent meta-analysis of the effects of biomass smoke on COPD found that biomass smoke exposure was significantly associated with the risk of COPD (odds ratio (OR) = 2.44, 95% CI: 1.9, 3.33), compared to those not exposed to biomass smoke, and this OR was even higher when looking specifically at women (OR = 2.73, 95% CI: 2.28, 3.28) [9].

**Figure 2:**
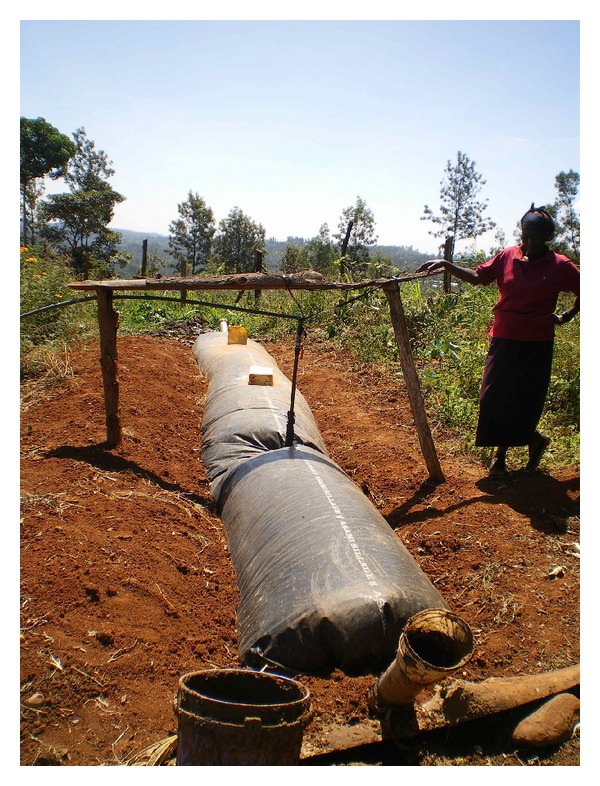
Biogas digester installed on a rural Kenyan smallholder dairy farm.

A report by the US Department of Energy on biomass cook stoves suggests that a 50% reduction in fuel use and a 90% reduction in emissions, relative to baseline technology, are important targets in global improvements to cook stoves in these environments [10]. Biogas digesters represent an important and accessible technology that produces biogas, an alternative fuel source consisting primarily of methane, which has the potential to reduce reliance on wood fuel use in cookhouse operations [11]. Biogas digesters are installed outside the cookhouse and function to anaerobically decompose organic material, such as livestock waste, to generate the gas which is then piped into the house and used for cooking (Figure 2). Methane burns cleanly and at high temperatures, thus providing a sustainable and cleaner-burning alternative fuel source to wood [12]. However, there is limited scientific documentation of the impact of biogas fuel use on spirometry outcomes and self-reported respiratory health outcomes.

The objective of this study was to assess the impact of biogas, as an alternative source of cookhouse fuel, on self-reported respiratory symptoms and spirometry outcomes for Kenyan women living on smallholder dairy farms.

## 2. Methods

### 2.1. Study Population

All participants included in the study lived on member dairy farms of Wakulima Dairy Limited (WDL), located in the Mukurweini area of central Kenya. Thirty-one farms were identified on which biogas digesters had been installed recently (between 3 and 24 months prior to the study, with assistance from a nongovernmental organization called Farmers Helping Farmers (FHF) [13]). Thirty-one referent farms without biogas digesters from the region were randomly selected from a list created using a chain referral sampling method [14, 15]. The sampling frame list of nonbiogas digester farms was created using referrals from farm occupants with digesters, matched to the biogas farms, based upon similar age of the participant, family size, and number of cows. In the event that a selected nonbiogas digester family chose not to participate in the survey, the next family on the list was contacted. 

### 2.2. Data Collection

Data collection took place in June and July 2010. Face-to-face interviews about the respiratory health of participants and of their children were conducted by a researcher at the women's houses, in Kikuyu (local language), with the aid of a translator. Questions on respiratory symptoms were based on the relevant questions from previously validated questionnaires such as the British Medical Research Council [16–18] in order to obtain externally validated responses regarding respiratory symptoms from the study population. The questionnaire was pretested and refined with the support of Kenyan women living on Prince Edward Island.

The study population also underwent a standard respiratory exam, including auscultation, palpation, and pulse oximetry, which was conducted at a common community venue by two 4th year University of Prince Edward Island (UPEI) nursing students who were supervised on-site by a critical care and emergency nurse (also from UPEI). Spirometry assessments were conducted by the critical care and emergency nurse using a Grace Medical Koko Legend Portable Spirometer (Grace Medical Marketing Inc., 5004 Barnwood Terrace, Kennesaw, GA, USA) according to the American Thoracic Society protocol [19], set to the Knudson-predicted values [20]. Clinical assessors were blinded to whether or not women lived on farms with biogas digesters.

### 2.3. Data Analysis

Each variable was evaluated for normality, graphically and using the Shapiro-Wilk test [21], with application of square-root transformations, where necessary, to transform for normality. Descriptive statistics were calculated (e.g., means, 95% confidence intervals, percentiles, etc.). For univariable analyses, standard unpaired *t*-tests, testing for equal variance according to Levene's test, and chi-square tests were applied to compare variables between the biogas and referent groups (significance at *P* ≤ 0.05).

Multivariable linear regression was used to determine the important predictors of spirometry outcomes for the participants. Variables obtained from univariable analysis were retained for model-building if *P* ≤ 0.2. Potential explanatory variables were tested for collinearity, and a possible causal diagram was also created for the potential explanatory variables to avoid the inclusion of intervening variables during the model-building process [22]. Forward selection (nonautomated) was used to determine the main predictors of the outcome in the model. Variables were initially included in the model-building process at a significance level of *P* ≤ 0.1. Intervening variables were then removed, and only main effects were retained in the final model at a significance level of *P* ≤ 0.05.

Linearity between predictor variables and spirometry outcomes was assessed using a scatter plot, with a Lowess-smoother line fitted to the plot. Scatter plots of the standardized residuals and predictor variables were also generated to test goodness-of-fit. Constant variance was assessed using the Cook-Weisberg test for heteroscedasticity, and influential observations were assessed using Cooke's distance. All participant data were analyzed using Stata/IC 11.1 for Mac (StataCorp 4905 Lakeway Drive, College Station, TX, USA).

This study was conducted according to the ethical guidelines established by the Canadian Tri-Council Guidelines for Involvement of Human Subjects in Research published by the National Sciences and Engineering Research Council, the Social Sciences and Humanities Research Council, and the Canadian Institute for Health Research. This project was reviewed and approved by the Dalhousie University Health Sciences Research Ethics Board prior to implementation.

## 3. Results

### 3.1. Population Characteristics

A comparison of the sociodemographic and physical characteristics of the biogas and referent groups revealed similar profiles for the two groups (Tables 1(a) and 1(b)). Women in the biogas group were slightly more likely to have a husband who was employed (74%) compared to the referent group (50%), though this was not significant at *P* ≤ 0.05. None of the participants in either group were smokers, although a few women in each group had other family members that smoked outside of the house or cookhouse buildings, but this difference was also not significant. The women in both groups had similar family sizes and numbers of cows (by design), and comparable cookhouse sizes, as well as similar ventilation mechanisms in their cookhouses [23]. Mean physical measures of the women were not significantly different between groups, although women with biogas digesters were slightly heavier and had slightly faster resting pulses compared to women in the referent group.

### 3.2. Self-Reported Respiratory Health

Women were asked about their respiratory status for the last six months as part of the questionnaire. Responses were initially recorded as 5-level categorical variables (every day, week, month, 6 months, or never) but were collapsed to binary (never/ever) variables for the analyses (Table 2). Women from referent farms were slightly more likely (*P* < 0.15) to have experienced difficulty breathing, shortness of breath, and chest pain while breathing, during the last 6 months, compared to women living on farms with a biogas digester. Women in the referent group were significantly more likely to report having any breathing problem, during the last 6 months, compared to the women with biogas digesters (*P* = 0.03).

The biogas group participants were also asked questions to assess their self-perceived changes in health after the installation of biogas digesters (Table 3). The majority of women reported improvements in general health, as well as the health of their children, reductions in wood smoke inhalation for themselves and their children, and reductions in respiratory problems for themselves.

### 3.3. Respiratory Assessments

After the interview, each participant underwent a respiratory assessment that involved spirometry and a standard respiratory exam, including auscultation and palpation. There were no clinical or statistically significant differences in the respiratory exam components between the two groups of women for measures such as inspiratory and expiratory wheezes, tracheal deviation, accessory muscle use, equal respiratory expansion, and pain or tenderness while breathing. 

The measured values for all spirometry outcomes were numerically lower than the predicted values, for women of that age, ethnicity, weight, and height, for both groups, with the exception of the ratio of FEV_1_/FVC (Table 4). Spirometry tests showed slightly better lung function in the biogas group than the referent group, but differences were not statistically significant.

The regression analyses found that the variation in respiratory outcomes could not be predicted by the variables included in the survey or respiratory exam, with the exception of FEV_1_. A multivariable linear regression model (Table 5) shows that both family size and milk income explained approximately 16% of the variation in FEV_1_  (*R* ^2^ = 0.16). For every increase in family size (by one member), there was an average reduction in the percent predicted FEV_1_ in the women of 0.02, after adjusting for milk income. Table 5 also shows that women with a monthly milk income of ≥5000 KSH experienced, on average, a reduction of percent predicted FEV_1_ of 0.07, compared to women living on farms with an average monthly milk income of <5000 KSH, after controlling for family size. The final model did not include whether or not the farm had a biogas digester (*P* > 0.05). Goodness-of-fit tests demonstrated that the model fits the data, and there were no outliers or influential observations. 

## 4. Discussion

Biogas digesters, which generate predominately methane, have been shown to be suited for introduction into remote agricultural regions of low-income countries [24–26]. A study by Xiaohua et al. [27] showed that biogas digesters, used in different regions of rural China, reduced the use of biomass fuel by 40% [27]. Zhang and Smith [28] also observed that, in certain situations where animal manure and water are in sufficient quantity, biogas digesters can reduce reliance on wood, leading to reduced exposure to wood smoke, and potentially to a reduction in negative respiratory outcomes related to wood smoke exposure [28]. To our knowledge, this study is the first that specifically demonstrates the potential for respiratory health benefits of biogas digesters as an alternative fuel source for poorly ventilated cookhouses on rural Kenyan smallholder dairy farms. This contrasts with the substantial evidence that exists relating improved cook stove designs and ventilation system (chimney) use with improved indoor air quality and improved health for women and children in low-income countries [29–33].

The introduction of biogas digesters into 31 member dairy farms of the Wakulima Dairy Limited located in central Kenya provided this opportunity to assess the short-term impact on the respiratory health of nonsmoking women who are regularly exposed to wood smoke in cookhouses. No statistically significant differences were observed for sociodemographic factors, including marital status, family size, husband employment off the farm, highest level of education attained, and monthly income levels from selling milk (Tables 1(a) and 1(b)). Statistically significant differences were also not observed in the comparison of farm environment features, including number of cows and cookhouse size. These comparisons were somewhat limited by the small study sample size, which was even smaller for certain questions. For example, husbands were employed off the farm on 74% of the biogas group versus 50% of the referent group but there were only 23 and 26 husbands in the biogas and referent groups, respectively.

The comparison of physical characteristics of women in the two groups also revealed few differences (Table 1(b)). The biogas women were similar in relation to mean age, height, weight, blood pressure, respiratory rate, pulse per minute, and blood O_2_ saturation. In contrast, more women in the referent group reported respiratory symptoms such as shortness of breath, difficulty breathing, and coughing and/or chest pain while breathing, during the last 6 months, compared to women in the biogas group (Table 2). A significantly larger proportion of women in the referent group reported having any breathing problems, in the last 6 months, compared to the group with biogas digesters (*P* = 0.03). The prevalence of reported chest pain in the referent group (35%) was double the prevalence in the biogas group (17%). Similarly, the prevalence of shortness of breath reported among the referent (52%) group was nearly double the prevalence in the biogas group (30%) (Table 2). Interpretation of this self-reported information is limited by the possibility for recall and reporting bias among participants, potentially leading to overestimations of the self-reported benefits. It was not possible to obtain baseline measurements from the biogas group prior to installation of the digesters, which would have reduced this limitation. Furthermore, the relatively small study population limited the statistical power to test for these differences, and hence only borderline significance for these observations was observed.

Assuming that report bias was negligible, the large differences in respiratory health symptoms were likely attributable to the women in the referent group having higher rates of wood smoke exposure, evident through the fact that they were spending, on average, 120% more time exposed to wood smoke while cooking per week, compared to women in the biogas group [23]. Studies investigating fuel use associated with improved stove interventions (not biogas digesters) in rural Mexican and Guatemalan cookhouses have shown that reducing biomass fuel use and wood smoke exposure also resulted in women reporting fewer respiratory symptoms such as wheeze, cough, difficulty breathing, and phlegm production [30, 34], supporting the findings from this study. The installation of improved stoves in the Guatemalan study resulted in a reduction of reported respiratory symptoms for the participants (OR = 0.7, 95% CI: 0.5, 0.97) [30], while the Mexican study showed that 37% of biomass-reliant women reported currently having a cough and 46% reported having a wheeze, compared to 21% and 30% in referent women, respectively [34]. 

One study from a randomized stove intervention trial in Guatemala investigated the self-rated health improvements for women, after installation of the improved wood stove for the intervention group [29]. The authors of that study found that approximately 53% of the women reported improvements in their health after the installation of the intervention stove [29]. In our study, 87% of women reported that their health improved after the installation of the biogas digester. Ninety-seven percent of the biogas group participants in our study also reported inhaling less smoke while cooking, and 52% reported having fewer respiratory problems (Table 3). However, the Guatemalan study was able to collect data before and after intervention, while our study relied on collecting information solely after the installation of the biogas digester. 

Spirometry is widely used in the diagnosis of COPD and other respiratory diseases in North America. However, spirometry is not normally performed or widely available in most African countries, and the lack of access to equipment and training means that there is a deficiency of baseline data for most African populations [35]. Due to this lack of spirometry data, there are currently no African standards for predicted spirometry measures. However, Glew et al. (2004) have shown that in these cases, where no predicted measures for African comparison populations exist, the corresponding African-American standards can be used [36]. Nevertheless, this African-American standard may have potentially introduced measurement error in our data.

Results from the spirometry testing showed that all measures taken for Kenyan farmwomen were lower than the predicted values for a similar population of African-Americans, with the exception of the ratio between FEV_1_/FVC (Table 4). However, none of the percent-predicted values was below 80%. The Global Initiative for Chronic Obstructive Lung Diseases (GOLD) reports that a percent-predicted FEV_1_ value ≥80% and a FEV_1_/FVC ratio >0.7 provides no indication of COPD presence [37]. According to these standards, average lung function measures for our study participants were within the normal range. The spirometry results observed in our study are also in accordance with results presented from the Guatemalan randomized stove intervention trial. The Guatemalan study also showed that none of the participants had spirometry results indicative of COPD, and significant differences in lung function between intervention and control groups were not observed [30]. 

Results from the multivariable linear regression model for FEV_1_ showed that both family size and milk income predicted approximately 16% of the variation in the spirometry outcome (Table 5). A larger family size increases general workload as well as increases the amount of time spent cooking, potentially resulting in decreased FEV_1_ measures for the women. The participants with milk incomes ≥5000 KSH were significantly older (*P* = 0.05) than the participants with lower milk income, by 5 years, on average. Therefore, age was a correlate of milk income; these data suggest that older women would have had greater cumulative lifetime exposures to cookhouse environments, which would not have been mitigated by the short-term exposure time for those with the biogas digester intervention.

It is possible that the limited duration between when the biogas digesters were installed and when spirometry testing was conducted was insufficient for improvements in pulmonary function to develop. Also, the biogas digesters were installed before the initiation of this study, so a before and postcomparison was not possible. Consequently, the pre-installation respiratory health of the women in the biogas group was not known. Also, the amount of time that elapsed since the installation (between 3 and 24 months) of the biogas digester until the data collection period (June/July 2010) was variable.

The participants continued to supplement biogas fuel with wood fuel in situations where insufficient amounts of biogas were produced by the biogas digester, or when cultural traditions impeded biogas use (e.g., cooking the traditional dish, Githeri, with wood). The amount of wood consumed per day was lower by 40% for the group of women using biogas digesters (average consumption of 14 lbs/day) compared to the referent group (25 lbs/day) [23]. Despite the lower wood consumption among women in the biogas group, the continued exposure to small amounts of wood smoke may have contributed to no statistically significant differences in measured respiratory function between groups. 

Physical measurements of wood combustion products such as particulate matter (PM), which has been linked to adverse respiratory health [38], were not collected for this study. Consequently, we are unable to compare environmental measurements among the biogas group participants (who had some continued wood smoke exposure) versus the referent group. It is possible that having ongoing exposure to wood smoke, at lower concentrations, may minimize or delay positive respiratory benefits attributable to the initial reduction in wood smoke exposure due to the biogas digester use. Clearly, burning the gas from the biogas digesters would have provided substantial improvements in particulate matter exposure in the biogas group compared to the referent group, and perhaps even compared to the modest improvements in particulate matter exposure purported for improved biomass stoves [10].

Certain language and cultural factors may also have affected the data collection during this study. All the women participating in this study communicated primarily in a local language called Kikuyu. Consequently, the nurse conducting all spirometry testing had to work through a translator who was not familiar with the spirometry equipment. As a result of the language barrier, some difficulties arose in explaining to the participants the instructions of how to properly complete the spirometry tests. For example, terms such as inspiration and expiration did not translate into Kikuyu; this language has a limited number of ways of saying “breathe.” In future study, a translator with medical knowledge would likely be beneficial. Future studies may also benefit from analysis of sputum for evidence of inflammatory cells and mediators, something not conducted in the current study.

It is also important to note that this technology for reducing wood smoke exposure is not suitable for all situations. The methane production and wood fuel reduction depend on the availability of organic material (such as cow manure) and water required for the biogas digester to operate. This type of small-scale biogas digester may be most ideally suited to family farms, with enough livestock to support the digester, in areas where chronic water shortages are not a concern [11]. A study by Walekhwa et al. [26] has shown that in, Uganda, uptake of this technology was dependent on the age and sex of the head of the household, the household income, the number of cows, the family size and the cost of traditional fuels (wood) [26]. The feasibility of greater adoption of this technology may be dependent on resource availability, as farmers in rural areas of developing countries may not have sufficient income to cover the initial capital costs required for biogas digester installation.

## 5. Conclusions

This study provides evidence that having a biogas digester is associated with improved self-reported respiratory symptoms among women living on rural Kenyan smallholder dairy farms. The majority of women living on biogas farms reported significant improvements in general health, inhalation of wood smoke, and reduction of respiratory problems, which they attributed to the use of the biogas digester. None of the spirometry results, for either groups of women, indicated any presence of chronic obstructive pulmonary disease. Spirometry tests showed slightly better lung function in the biogas group, but differences were not statistically significant, likely due to the limited sample size and short time between biogas installation and spirometry testing. Further investigation, with greater numbers of participants having well-functioning biogas digesters for a longer period of time, would be beneficial to determine more thoroughly the effects of biogas digesters on respiratory health for Kenyan farmwomen.

## Figures and Tables

**Table:** (a)

Variable	Biogas farms		Referent farms	
	*n* = 31		*n* = 31	
Sociodemographic characteristics	Percent (%)	Number	Percent (%)	Number
Currently pregnant	0	0	6	2
Employed off the farm	39	12	35	11
Currently married	74	23	84	26
Husband employed off the farm	74^§^	17^§^	50^±^	13^±^
Current smoker	0	0	0	0
Highest level of education attained				
None	6	2	10	3
Standard 4	13	4	6	2
Standard 8	48	15	61	19
Form 4	19	6	19	6
Technical college	13	4	3	1
Monthly income from selling milk				
<5000 KSH	61	19	68	21
5000–10 000 KSH	32	10	23	7
>10 000 KSH	6	2	10	3

No differences exist between the groups at significance of *P* ≤ 0.05.

^§^based on *n* = 23 husbands; ^±^based on *n* = 26 husbands.

KSH: Kenyan shilling.

**Table:** (b)

	Biogas farms				Referent farms			
Variable	*n* = 31				*n* = 31			
	Mean	95% CI	Median	Range	Mean	95% CI	Median	Range
Farm environment								
Family size (number of people)^1^	3.5	2.9, 4.2	3	1, 7	3.9	3.3, 4.5	3	1, 8
Number of cows^2^	3.7	3.0, 4.4	3	2, 12	3.3	2.7, 4.1	3	1, 10
Cookhouse size^1^ (m^3^)	20	18, 23	21	6.5, 32	19	16, 22	18	8.4, 37
Physical characteristics								
Age^1^	45	42, 49	45	22, 63	44	40, 48	44	24, 72
Height (inches)^1†^	63	62, 64	63	59, 71	62	61, 63	63	57, 66
Weight (lbs)^1†^	156	144, 168	152	119, 255	145	135, 154	144	98, 214
Blood pressure^1†^								
Systolic	128	123, 134	128	108, 180	126	121, 130	122	108, 160
Diastolic	84	80, 88	82	62, 118	83	80, 86	84	68, 100
Respiratory rate per minute (at rest)^1†^	18	17, 19	18	15, 24	19	18, 19	18	16, 22
Pulse per minute (at rest)^1†^	83	78, 88	83	57, 110	77	72, 81	73	49, 104
Blood O_2 _saturation^1†^	97	96, 97	97	94, 100	96	95, 97	96	88, 100

No differences exist between the groups at significance of *P* ≤ 0.05.

^1^Parametric tests performed on raw (normally distributed) data.

^2^Parametric tests performed on square root transformed data; means, 95% CIs, medians and ranges presented were back-transformed.

^†^ *n* = 30—one participant (biogas group) was not able to attend the respiratory clinic.

**Table 2:** Comparison of participant responses related to self-reported health for the biogas and referent groups of Kenyan farmwomen.

	Biogas farms		Referent farms		
Variable	*n* = 30		*n* = 31		*P* value
	Percent (%)	Number	Percent (%)	Number	
Current cough	26	8	39	12	0.36
Frequent coughing (during last 6 months)	19	6	26	8	0.59
Shortness of breath (during last 6 months)	30	9	52	16	0.09
Breathing difficulty (during last 6 months)	23	7	42	13	0.12
Chest pain with breathing (during last 6 months)	17	5	35	11	0.10
Uses medication to help breathe (during last 6 months)	3	1	3	1	0.98
Any breathing problem (during last 6 months)^1^	43	13	71	22	0.03

^1^Reported having any of shortness of breath, breathing difficulty, and/or chest pain while breathing.

**Table 3:** Responses to questions about perceived changes in personal and child respiratory health due to biogas digester use, from women in the biogas digester group.

Variable	No change	Agree
	Percent (number) *n* = 31	
Health is better	13 (4)	87 (27)
Breathe in less smoke	3 (1)	97 (30)
Fewer breathing problems	48 (15)	52 (16)
	Percent (number) *n* = 18	
Children's health is better	28 (5)	72 (13)
Children breathe in less smoke	22 (4)	78 (14)
Children have fewer breathing problems	50 (9)	50 (9)

**Table 4:** Descriptive statistics of spirometry measures for the biogas and referent groups of Kenyan farmwomen.

Variable	Biogas farms (*n* = 30 women)			Referent farms (*n* = 31 women)			*P* value
	Mean	Median	Range	Mean	Median	Range
FVC							
Measured	2.6	2.7	1.4, 4.3	2.7	2.6	1.6, 4.1	0.65
Predicted	3.1	3.2	2.1, 5.5	3.0	3.1	2.2, 3.6	0.39
Percent predicted (%)	84	85	49, 114	89	85	51, 131	0.16
FEV_1_							
Measured	2.3	2.3	1.3, 3.9	2.3	2.3	1.6, 3.1	0.92
Predicted	2.6	2.6	1.8, 4.4	2.5	2.6	1.8, 2.9	0.51
Percent predicted (%)	90	89	61, 120	93	93	60, 117	0.34
PEFR							
Measured	5.2	5.0	2.8, 7.4	4.9	4.7	2.9, 8.3	0.28
Predicted	6.1	6.0	4.9, 10	5.9	6.0	5.0, 7.3	0.36
Percent predicted (%)	87	87	47, 120	83	84	47, 133	0.48
FEF_25–75_							
Measured^1^	3.2	3.2	1.5, 5.3	3.1	2.9	2.1, 5.8	0.94
Predicted^1^	3.5	3.5	2.6, 5.4	3.4	3.4	2.6, 4.0	0.47
Percent predicted (%)	91	95	47, 120	93	88	59, 161	0.78
FEV_1_/FVC ratio							
Measured	0.90	0.89	0.76, 1.0	0.89	0.90	0.76, 1.0	0.72
Predicted	0.85	0.85	0.83, 0.99	0.86	0.85	0.82, 0.99	0.86
Percent predicted (%)	105	107	90, 117	104	104	88, 120	0.70

^1^Parametric tests performed on square root transformed data.

FVC: forced vital capacity; FEV_1_: forced expiratory volume at 1 second.

PEFR: peak expiratory flow rate; FEF_25–75_: forced expiratory flow during the middle 50% of the expiration.

Percent predicted values calculated based on Knudsen reference values for African-American populations.

**Table 5:** Multivariable linear regression model of percent predicted FEV_1_, for Kenyan farmwomen.

Variable	Coefficient	Standard error	*P* value	95% CI
Family size	−0.02	0.01	0.02	−0.04, −<0.01
Milk income				
<5000 KSH	referent	—	—	—
≥5000 KSH	−0.07	0.03	0.03	−0.14, −0.01
Constant	1.02	0.04	<0.01	0.95, 1.10
